# Books Reviewed

**Published:** 1863-09

**Authors:** 


### BOOKS REVIEWED.

*A Treatise on Hygiene with special reference to Military Service. By* Wm. A. Hammond, M. D., *Surgeon-General U. S. Army; Fellow of the College of Physicians of Philadelphia; Member of the Philadelphia Pathological Society; of the Academy of Natural Science; of the American Philosophical Society; Honorary Corresponding Member of the British Medical Association; Member of the Verein Fur Geminschaftliche Arbeiten Zur Furderung Der Wissenshaftlichen Heilkunde; late Professor of Anatomy and Physiology in the University of Maryland; late Surgeon to and Lecturer on Clinical Surgery at the Baltimore Infirmary, etc., etc. Philadelphia:* J. B. Lippincott & Co., *Nos.* 715 *and* 717 *Market street,* 1863.

The reasons for presenting this treatise to the profession are so well expressed in the author's own words that we take the liberty to quote from the preface, since it will show the object and design of the work better than any other description:

"If I had not believed that a great necessity existed for a treatise upon some of the principal subjects of hygiene, I certainly should not, in addition to my onerous public duties, have undertaken the task of preparing the present volume. That a growing attention to the subject of sanitary science is being manifested, cannot be doubted. The most intelligent members of the medical profession recognize the principle that their efforts should be directed more especially to the prevention of disease than to its cure, and the people, who are rarely slow to comprehend matters which it is to their advantage to know, are beginning to appreciate the same fact.

But while I do not wish to be understood as at all doubting the efficacy of proper medication in the treatment of disease, I am sure that the curative influences of hygienic measures have been too much neglected, and that drugs, the traditional actions of which have been positively disproved by physiological and chemical researches, as well as by the soundest deductions from pathology, are too frequently administered through a strict adherence to the routine which hinders the development of medical science, and cramps the powers of those who labor for its advancement. One object therefore which I had in view, was to lay before the profession and those who contemplate entering it some of the principal facts which bear upon the hygienic condition of man in causing, preventing, and curing disease.

But I had a still stronger motive to actuate me. The nation had entered upon a war, for the preservation of its liberties, the most gigantic ever undertaken in the history of the world. Hundreds of thousands, from the boy to the old man, had devoted themselves to the service of their Country—men whose value to the State could not be estimated, and upon whom its future greatness, both in war and peace, in a great measure depended. Thousands of physicians had been found to take the medical charge of the armies created—many of them well known for their professional eminence, and others, by far the greater number, young and inexperienced, though not lacking the will and the ability to do their whole duty when that duty was pointed out to them. Many of these latter have now become fully equal to the laborious service to which they have devoted themselves, and each month adds efficiency and distinction to the medical corps of the regular and volunteer forces of the army.

In the military service, more than any other, a knowledge of the means of preventing disease and of facilitating recovery by methods other than the mere administration of drugs is necessary. Armies are often so situated that their salvation depends upon the knowledge which the medical officer may possess, and it never happens that some important application of hygienic principles cannot be made to them by those who are charged with their medical superintendence.

But though many excellent treatises upon individual hygiene are to be met with in the French and German languages, there is not one to be found in the English tongue. The little book of Dr. Pickford does not profess to go at any length into the subject, and is devoted almost entirely to the consideration of the meteorological influences exerted upon health, and to the discussion of points of public hygiene; and the excellent treatise of Prof. Dunglison has for many years been out of print. As to military hygiene, I know of no other book on the subject, in the English language, than the capital little manual of Prof. Ordronaux, of Columbia College, which, though containing many most valuable hints in regard to the health of the soldier, was not intended by its accomplished author as a treatise on the subject.

There was no work then to which I could refer those who came to me for information which I had no time to give them as fully as was desirable; and as I had for several years given a large portion of my leisure to the study of hygiene—rather, however, in a desultory way than with any systematic objects in view—I concluded to devote the hours which would otherwise have been passed in rest, in preparing a volume upon the more important subjects belonging to the science of hygiene, especially those which have a bearing upon military service.

It is not pretended that this volume is complete. There are several subjects other than those considered, such as Occupation, Exercise, the Excretions, Marriage, Celibacy, etc., which I would have been glad to have taken up, had I not been convinced that the need for some work on sanitary matters was imperative; and therefore, notwithstanding the imperfect result of my labors—the shortcomings of which no one can perceive more clearly than myself—I have concluded to stop for the present, and to defer to a second edition, should such be called for, the more complete fulfillment of my task, by the consideration of topics not only interesting in themselves, but important in their bearings upon the health, the comfort, and the happiness of mankind.

Moreover, I have been restrained from expressing my views fully upon some subjects, for the reason that the immense amount of material which has been collected in the Surgeon-General's office during the past year—an amount unprecedented in the annals of military medicine and surgery, and more even than is contained in the published medical records of all the armies of the world—is not as yet so arranged as to be in a form for satisfactory study, and I therefore preferred, both for my own sake and that of the reader, to delay the consideration of points which otherwise I should have discussed with insufficient light. Besides, much important information might have been given in regard to the relations of medical statistics to hygiene, but for the fact that the associated matter would have been in many instances of value to the enemy in a military point of view.

Since this treatise was commenced, events have been developed with surprising rapidity, and, in consequence, several subjects in regard to which opportunities for forming definite opinions had not been afforded, are now matters of fact. Such, for instance, is that of the adaptability of the negro race for all the purposes of war, which, at the time the chapter on Race was written, was, in some respects, an open question, but which has been recently shown to be no longer a subject of doubt. The opinion then expressed relative to the immunity of this race to attacks of malarious diseases has received additional confirmation from the official reports which have recently come to hand, from which it appears that while the white troops are affected to the extent of 10·80 per cent. with diseases of malarious origin, the negro troops serving in the same army show only 0·80 of such diseases.

It is only by yielding our opinions to the necessities of the age in which we live, when every science bearing upon medicine is being developed by the labors of thousands of investigators, that we can claim the right to be regarded as wise physicians seeking only the good of those committed to our charge, rather than our own personal advantage. In science we believe nothing until it is proven, and even then we should be ready to forsake our most cherished doctrines when the evidence of their instability is forthcoming. If, therefore, I have been positive in the expressions which are at variance with those held by others, it is only because I *now* believe them to be correct. To-morrow I may renounce them all.

But even in my positiveness, I hope I have not forgotten the proprieties of life, or the forbearance and courtesy which should prevail in all discussions, especially in those of a scientific character."

The work contains forty-three chapters, under the following heads:

"General Qualifications and Disqualifications of Recruits—Special Qualifications and Disqualifications of Recruits—Race—Temperaments in General — Particular Temperaments — Idiosyncrasy—Age—Sex—Hereditary Tendency—Habit—Morbid Habits—Constitution—The Atmosphere—The Accidental or Non-essential Constituents of the Atmosphere — Physical Properties of the Atmosphere—Temperature—Light—Electricity—Water—Soil—Locality—Climate—Acclimation—Habitations—Hospitals—Principles of Hospital Construction—Field Hospitals—Lighting of Hospitals—Heating of Hospitals — Ventilation of Hospitals — Barracks — Camps—Food—Alimentary Principles—Physiological and Sanitary Relations of Food—Animal Compound Aliments—Vegetable Compound Aliments—

Accessory Food—Alimentation of the Soldier—Clothing—The Hygienic Relations of Clothing with the several parts of the Body."

Since our readers will have much greater interest in what the Surgeon-General has to say upon the use of Alcohol as food, as what he calls accessory food, than in any other notice we can give of the work, we will quote some of his remarks upon that subject, though at the risk of occupying considerable space. At the present time, it is certainly a very important practical work, and the author shows himself thoroughly acquainted with his subject, and actively alive to the great interests he advocates and desires to promote; and while we can only copy a few of his remarks upon a single subject, we yet earnestly recommend the careful perusal of the whole volume. Especially should all the medical officers of the army become familiar with its facts and teachings; to them, and to those under their care, it is of vital importance:

"We see, then, that alcohol, like other substances, is absorbed into the blood, and exerts its influence on the system through the medium of that fluid. In the next place, we have to inquire relative to the effects which it thus produces.

"Pure alcohol is a violent poison. In the dose of less than one ounce I have seen it cause death in a medium-sized dog, and many cases are on record of fatal effects being immediately produced in the human subject after comparatively small quantities had been swallowed. When diluted, its effects are not so rapidly manifested, and in this form, when taken in sufficient quantity, the condition known as intoxication is produced. Previous to this point being reached, the nervous and circulatory systems become excited, the mental faculties are more active, the heart beats fuller and more rapidly, the face becomes flushed, and the senses are rendered more acute in their perceptions. If now the further ingestion be stopped, the organism soon returns to its former condition without any feeling of depression being experienced; but if the potations are continued, the complete command of the faculties is lost and a condition of temporary insanity is induced. If further quantities are imbibed, a state of prostration follows, marked by coma and complete abolition of the power of sensation and motion. Such is a brief outline of the obvious symptoms which ensue upon the use of alcoholic liquors in considerable quantities. When taken in amounts less than are sufficient to induce any marked effect upon the circulatory and nervous systems, there is, nevertheless, an influence which is felt by the individual, and which is mildly excitatory of the moral and intellectual faculties.

"But besides these perceptible results of the use of alcoholic liquors, there are other physiological effects which flow from their use, far surpassing in importance any that have been named, and which mainly render the substances in question useful as aliments.

"We have already passed in review the principal phenomena connected with the retrograde metamorphosis of the tissues of the body. We know that a certain amount of tissue is decomposed with every functional action of the organ to which it belongs, and we at once perceive that, were it not for the formative processes which are going on, whereby new material derived from the food is deposited, to take the place of that which is removed, death would very soon result. It is often important to arrest this destruction of tissue, without at the same time lessening the force which would otherwise be derived from its continuance; or it may be desirable to obtain a great amount of force from an individual in a limited period. In alcohol we have an agent which, when judiciously used, enables us to accomplish both these ends, together with others scarcely less important, which will be alluded to more at length hereafter. The operation of alcohol will be best illustrated by an example.

"Let us suppose that a plowman, laboring twelve hours a day, upon a diet consisting of ten ounces of meat and sixteen of bread, finds that he loses weight at the rate of one ounce per day. Now, in order to preserve his life, he must either take more food or he must lessen the waste of his tissues. Meat and bread are both expensive, and he finds it difficult to obtain them, or, what is not at all improbable, the quantity he eats is as much as he has any appetite for. The alternative which presents itself to him is that of working less. If he is his own master, this would be a very excellent way of getting rid of the difficulty. He would shorten the period of his labor to ten hours, and then, instead of losing weight, he would perhaps gain an ounce a day. But it may happen that this alternative is not open to him—he must work twelve hours a day. In this condition of affairs he takes a mug of porter or a glass of wine, or what would be worse, a dram of whisky, after his mid-day meal. He finds that he is pleasantly exhilarated, his vigor is increased, and he labors on to the close of his task contentedly, and when it is concluded, is in better spirits and less fatigued than he has been before when his day's work was ended. He returns to his home, and, on weighing himself, finds that he has lost but half an ounce. He repeats his beverage the next day; like results follow, and, when he weighs himself, he ascertains that he has lost nothing. The inference therefore is, that the beverage he has imbibed, or some constituent of it, has retarded the destruction of his tissues, and has itself aided in supplying the material for the development of the force he has exercised in his labor.

"Now it may be supposed that this is altogether a fancy picture, that it is a theory based upon assumptions only, like too many others which encumber science. In physiology or hygiene we believe nothing but that which is demonstrated, and even then we do so provisionally, with the full understanding in our minds that if to-morrow new facts are brought forward which appear to be inconsistent with those upon which a favorite theory rests, and which are of greater weight, the hypothesis shall be abandoned without hesitation. Let us see, therefore, what evidence we have to support the view that alcohol retards the destruction of the tissues and supplies material for the generation of force.

"Many years ago, Dr. Prout ascertained that after the use of alcohol the amount of carbonic acid ordinarily excreted by the lungs became considerably reduced. Within the past few years other investigators have arrived at similar conclusions, and have extended their inquiries to the other excretions of the system. Thus Böcker ascertained that under the use of alcohol not only was the amount of carbonic acid exhaled by the lungs lessened, but there was a very decided diminution in the quantity of urine eliminated and in the amount of its solid constituents.

"My own experiments tend to the same general conclusions as those of Böcker. They had reference to the influence of alcohol when the food was just sufficient for the wants of the organism, when it was not sufficient, and when it was more than sufficient. Four drachms of alcohol were taken at each meal, diluted with an equal quantity of water.

"During the first series, when the food was of such a character and quantity as to maintain the weight of the body at its normal standard, I found, as the result of experiments continued through five days, during which time 60 drachms of alcohol had been taken, that the weight of my body had increased from 226·40 pounds to 226-85 pounds, a difference of ·45 of a pound. In the same period, the amount of carbonic acid and aqueous vapor exhaled from the lungs had undergone diminution, as had likewise the quantity of urea and its solid constituents.

"During these experiments my general health was somewhat disturbed. My pulse was increased to an average of ninety per minute, and was fuller and stronger than usual, and there was an indisposition to exertion of any kind. There were also headache and increased heat of skin.

"The inference to be drawn from these experiments certainly is that, when the system is supplied with an abundance of food, and when there are no special circumstances existing which render the use of alcohol advisable, its employment as an article of food is not to be commended. But there are two facts which cannot be set aside, and these are, that the body gained in weight and that the excretions were diminished. These phenomena were doubtless owing to the following causes: First, the retardation of the decay of the tissues; second, the diminution in the consumption of the fat of the body; and third, the increase in the assimilative powers of the system, by which the food was more completely appropriated and applied to the formation of tissue.

"The *quasi* morbid results which followed are just such as would have ensued upon the use of an excessive amount of food of any kind, or the omission of physical exercise when the body has become habituated to its use. If I had increased the extent of exercise taken, there is no doubt there would not have been the undue excitement of the circulatory and nervous systems that was manifested.

"The truth of these propositions is seen in the second series of investigations, during which the food ingested was such as I had previously ascertained involved an average decrease in the weight of the body of ·28 of a pound daily. Under the use of the alcohol, not only was this loss overcome, but there was an average increase of .03 of a pound daily. The effects upon the excretions were similar to those which ensued in the course of the experiments of the first series."

---

*Gastrotomy—Large Abdominal-Uterine Tumor, extirpated by* JOHN O'REILLY, M. D., F. R. C. S. I. *Reported by* RICHARD J. HALTON, L. R. C. S. I. *New York: Printed by* ROBERT CRAIGHEAD, 1863.

This is the report of a remarkable case of abdominal tumor successfully removed by operation. The following extracts are made from the pamphlet, which will give many of the facts and circumstances of the case, in the language of the author:

"Mrs. L., the subject of the present report, was a married woman about 55 years old, the mother of several children. Seven years ago the tumor first made its appearance, at that time causing little or no inconvenience; but as time passed it increased in size, and finally filled up the whole front of the abdomen, distending the parts so as to produce great deformity, while the pain became considerable, and latterly the constitution began to give way altogether. There was a good deal of emaciation, and the lower extremities showed considerable œdema. On examination, the tumor was found to be firmly fixed, but the parietes were movable slightly upon it. There was no evidence of fluctuation, but it was supposed, from its great elasticity before the operation, that it contained a number of cysts. As the patient was evidently sinking, Dr. O'Reilly determined to give her the chance the operation affords, a middling one, according to the general opinion of the profession; but by his theory of the physiological action of the organic nervous glands, and the effect of opium thereon, he calculated on being able to prevent *peritonitis*, which is so often fatal after these operations. With this intention, therefore, he prepared the patient, and the night before the operation gave her forty drops of laudanum, to keep her bowels quiet and *contracted* during the operation. She slept well, and in the morning, just before the operation, her pulse was 144. She was cheerful, perfectly easy, and very hopeful as to the result. On the first of July, at 12½ o'clock P. M., chloroform being administered by Mr. James O'Dowd, Dr. O'Reilly, in presence of Drs. Nelson, T. G. Thomas, and myself, commenced the operation. The first incision was carried from the umbilicus to the pubes, and the integuments, muscles, and peritoneum, being divided, the tumor was brought into view, and Dr. O'Reilly made an attempt to introduce a trocar, but without success: he tried in another spot with the like result. Indeed, from its feel, it was evidently solid; so he prolonged the incision almost to the ensiform cartilage, and now the parietes, contracted at each side, left the tumor exposed. It hid the *viscera* altogether, and when it came to be lifted out it was found to be attached to the uterus, or rather the uterus was attached to it behind, and the fallopian tubes embraced it on either side. The lateral ligaments were closely attached to it; in fact they formed an all but complete investment, for they stretched out on it apparently as the tumor increased. There was a great deal of difficulty in finding the exact attachments of the tumor, but the principal ones appeared to be from the third to the fourth lumbar *vertebra*, and then extending along the sacro-iliac synchondrosis of the right side—how far down in the pelvis may be imagined when I mention the fact that I tied a vessel low down in the recto-uterine space: the attachments were partly torn through with the hand, partly cut. The principal vessel entered the tumor opposite the third lumbar vertebra, and was of considerable size; there were about six smaller ones: each was tied as it was divided; nevertheless, hæmorrhage from the numerous oozing-points was considerable. When the tumor was removed, the abdominal cavity (the lower part of it) was sponged out several times to remove the blood. The edges of the wound were brought together first by a deep metallic suture, twisted, embracing the soft parts external to the peritoneum; and by a common interrupted suture securing the integuments, sticking-plaster being applied in the intervals between the sutures; and finally a towel folded flat, a pad of tow, and a many-tailed bandage, completed the dressing, the two last tails of the bandage being brought down under the thighs, up in front of the groin, and fastened there to guard against any slipping; and then the patient being thoroughly washed, and all traces of blood removed, was carried to bed. She got immediately a draught containing two grains of opium, and after half an hour, that having produced no apparent effect, she got two grains more. She was ordered to take nothing but a small piece of ice, sucked occasionally, and to take two grains of opium every third hour, making in all eight grains between the hours of one P. M. and nine P. M. She slept about three hours after the last dose of the opium, and had no pain of any kind. The opium to be continued during the night every third hour.

"It is worthy of remark that, during the operation, all rules laid down in books on the subject were entirely disregarded. The window was kept open, admitting a free draught. The intestines were allowed to come out, and they were never touched until the operation being over, when they were all lifted in again, Dr. Nelson well remarking that "the flannel usually employed to keep them in was as rough to the delicate covering of the intestines as a clothes-brush would be to the skin." Vomiting having occurred, the stomach was thrown forwards out of the abdominal cavity, as it were: its contractions and dilations could be plainly observed. The liver was quite healthy. The tumor, which might be called fatty fibro-cellular, displayed on section two large lobes of fat, intersected with fibrous bands, large sinuses (venous), connected by cellular tissue; it presented very much the appearance of a cow's udder, and weighed over *thirty pounds.* After the patient was got into bed, she expressed herself as being quite easy. She had suffered no pain; pulse 100; voice pretty strong."

We quote the record of the seventh day, which shows the termination of the case, so far as the operation is concerned:—

"July 7th.—She took four grains of opium during the night. The dressings were removed to-day, as well as the pins and sutures; the whole wound was healed by the first intention, with the exception of the lower part where the ligatures came out; there is not the slightest pain on pressure over any part of the abdomen, which is soft and relaxed; she expresses a wish to get a beef steak; she wants to know when she can get up and go home (she occupied a room at her sister's residence)."

Soon after the record was made, the patient took, upon her own responsibility, three ounces of cream of tartar, and died in consequence on the second day after the partaking of the cream tartar, and the ninth from the operation. Dr. O'Reilly remarks that—

"I am satisfied Mrs. L. might have lived some years had she *obeyed* my orders, and not, in *violation* of her promise, taken the cream of tartar.

It will be recollected that the fact of the cream of tartar acting as a purgative, proved the *non-existence* of peritonitis."

---

*The Pharmacopœia of the United States of America. Fourth Decennial Revision. By authority of the National Convention for revising the Pharmacopœia, held at Washington, A. D.* 1860. *Philadelphia:* J. B. Lippincott & Co. (Price $1,00.

This work contains the proceedings of the National Convention of 1860

for revising the Pharmacopœia, with preface and preliminary notices, together with the Materia Medica. In the catalogue of Materia Medica the names of medicinal substances are given in Latin and English, and synonyms in English are added, and a brief description of each article is generally introduced. The body of the work is devoted to the medicinal preparations; the composition and mode of manufacture is given, while therapeutical uses and the doses of the various compounds are omitted. This will be considered as a defect by those who would like to consult the work for the purpose of learning the composition, mode of manufacture, therapeutical uses, and dose. It is not, however, possible to include everything pleasant and profitable to know of materia medica and of the various medicinal preparations in a work of small size, which is cheap and convenient as a book of reference. For the physician who compounds his own remedies, it will be found exceedingly valuable, and it contains much which every physician will find it desirable to know. It has been corrected and revised until it may now be considered a perfect standard. It contains tables of medicines introduced into the Materia Medica and dismissed from it, of medicines introduced into the medicinal preparations and dismissed from them. It also has a table of the changes in the Latin officinal names, and of the changes in the position of medicines. It is a condensed and corrected Pharmacopœia of the United States, and commends itself to all those who would be guided by the most recent and approved standard.

